# Evolution of disease transmission during the COVID-19 pandemic: patterns and determinants

**DOI:** 10.1038/s41598-021-90347-8

**Published:** 2021-05-26

**Authors:** Jie Zhu, Blanca Gallego

**Affiliations:** Centre for Big Data Research in Health, UNSW Sydney, Sydney, NSW 2052 Australia

**Keywords:** Health policy, Public health, Diseases, Infectious diseases, Computational models, Data processing, Statistical methods

## Abstract

Epidemic models are being used by governments to inform public health strategies to reduce the spread of SARS-CoV-2. They simulate potential scenarios by manipulating model parameters that control processes of disease transmission and recovery. However, the validity of these parameters is challenged by the uncertainty of the impact of public health interventions on disease transmission, and the forecasting accuracy of these models is rarely investigated during an outbreak. We fitted a stochastic transmission model on reported cases, recoveries and deaths associated with SARS-CoV-2 infection across 101 countries. The dynamics of disease transmission was represented in terms of the daily effective reproduction number ($R_t$). The relationship between public health interventions and $R_t$ was explored, firstly using a hierarchical clustering algorithm on initial $R_t$ patterns, and secondly computing the time-lagged cross correlation among the daily number of policies implemented, $R_t$, and daily incidence counts in subsequent months. The impact of updating $R_t$ every time a prediction is made on the forecasting accuracy of the model was investigated. We identified 5 groups of countries with distinct transmission patterns during the first 6 months of the pandemic. Early adoption of social distancing measures and a shorter gap between interventions were associated with a reduction on the duration of outbreaks. The lagged correlation analysis revealed that increased policy volume was associated with lower future $R_t$ (75 days lag), while a lower $R_t$ was associated with lower future policy volume (102 days lag). Lastly, the outbreak prediction accuracy of the model using dynamically updated $R_t$ produced an average AUROC of 0.72 (0.708, 0.723) compared to 0.56 (0.555, 0.568) when $R_t$ was kept constant. Monitoring the evolution of $R_t$ during an epidemic is an important complementary piece of information to reported daily counts, recoveries and deaths, since it provides an early signal of the efficacy of containment measures. Using updated $R_t$ values produces significantly better predictions of future outbreaks. Our results found variation in the effect of early public health interventions on the evolution of $R_t$ over time and across countries, which could not be explained solely by the timing and number of the adopted interventions.

## Introduction

Mathematical and computational models of disease outbreaks are used to generate knowledge about the biological, behavioral and environmental processes of disease transmission, as well as to forecast disease progression. Public health responders rely on insights provided by these models to guide disease control strategies^1^. As an example, in mid March 2020, the British government changed its SARS-CoV-2 response policy following a brief on simulation results from Ferguson et al.^2^, which indicated an unacceptable forecasted number of deaths in the absence of more stringent control measures.

Different models have been applied to model the spatial and temporal dynamics of SARS-CoV-2 transmission (see the review study^3^). They range from simple deterministic population-based models^4–6^, that assume uniform mixing, to complex agent-based models^2,7^ in which individuals defined by different attributes related to their susceptibility, infectiousness and social interactions transmit the pathogen to each other, given rise to heterogeneous transmission patterns.

Irrespective of their complexity, the accuracy of these models is constrained by the validity of the epidemiological parameters that underpin them. For example, the model parameters controlling the risk of infection together with the social contact between infectious and susceptible individuals determine the transmission rate, which in turns influences the peak and duration of the epidemics. By manipulating these parameters, modelers can represent the impact of public health measures such as social distancing (lowering the contact rate) or wearing protective masks (lowering the risk of infection).

This study evaluates the effectiveness of initial public health interventions globally by estimating the effective reproduction number (the expected number of secondary infections resulting from an infectious individual) on a daily basis and highlights the importance of using updated reproduction numbers for outbreak forecast. In the second section, we describe the “Methods”. In “Results”, we first present the patterns of initial public health interventions and effective reproduction numbers for a fixed temporal window across countries. Then, we look into the temporal patterns of number of public health interventions, effective reproduction number and incidence counts using a time-lagged cross correlation analysis. Lastly, future outbreaks are predicted to illustrate how the currency of epidemiological parameters can influence the forecasting of expected infection counts. We end with a “Discussion”.

## Methods

### Data sources

**Figure 1:**
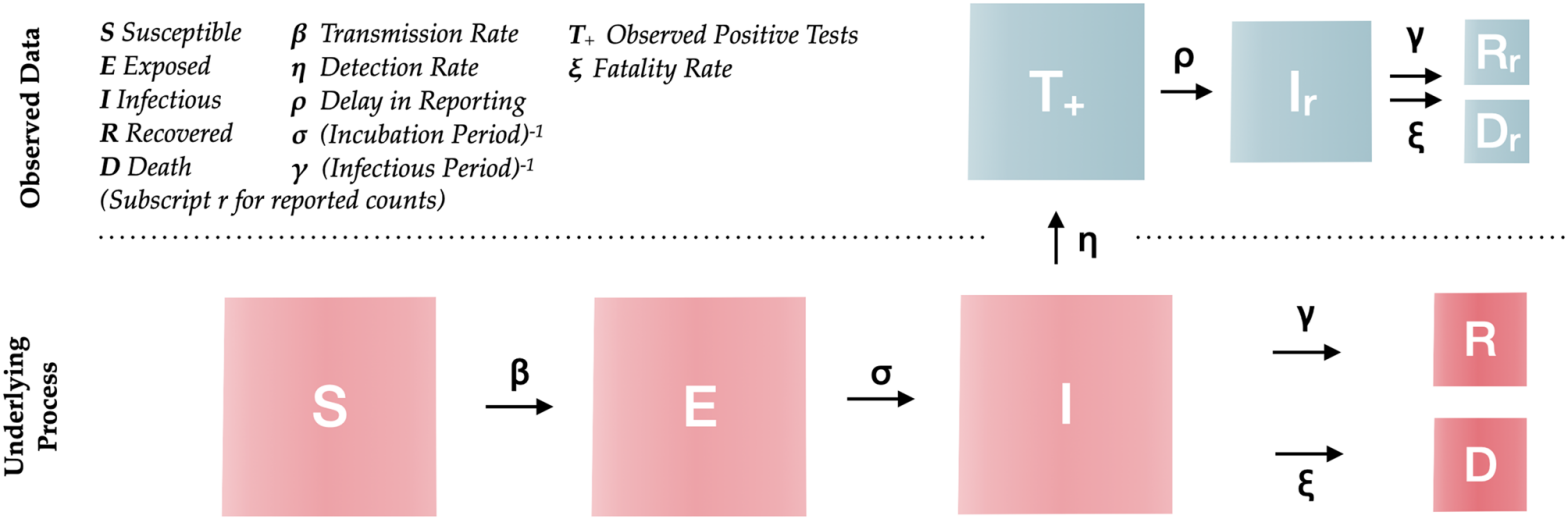
Simulation model structure. The population is divided into the following five classes: susceptible, exposed (non-infectious and asymptomatic), infectious (asymptomatic and symptomatic), removed (i.e., isolated, recovered, or otherwise non-infectious) and dead. The reported data is modeled with four classes: observed positive tests, reported infections, removed and deaths.

By 15 May 2020, there were 188 countries with recorded SARS-COV-2 infections, and 101 of them had implemented at least one public health intervention to contain the spread of the virus and had at least one death^8^. We modeled the transmission dynamics of SARS-COV-2 between 22 Jan to 15 May 2020 by fitting a stochastic SEIRD model^9^ (illustrated in Fig. 1) to three available time series: (1) daily number of incident cases, (2) daily number of deaths, and (3) daily number of recoveries, as recorded for each country by the Johns Hopkins Coronavirus Resource Center^10^.

To monitor public health interventions, we captured government stringent policies using the Oxford COVID-19 government response tracker^8^, which contains 17 policies organized into three groups: containment and closure policies, economic policies and health system policies. We explored 12 of these policies, namely those that have a more immediate and direct impact on individual’s lifestyle and well-being. Social distancing policies (including travel policies) were further classified into two levels depending on whether the government took a recommended or required stance. International travel controls were divided into four levels: (1) screening arrivals, (2) quarantine arrivals from some or all regions, (3) ban arrivals from some regions, and (4) ban on all regions or total border closure.

### Epidemic simulation model and analysis of transmission patterns

We built an extended version of a SEIRD model that included transitions between reporting states and disease states in order to account for delays in reporting (Fig. 1). The model incorporates uncertainty in reported counts by explicitly modeling a Poisson process of daily reported infection, recovered and death counts, as well as of tested individuals. Following previous literature^11,12^, the mean incubation period was assumed to be Erlang distributed with mean 5.2 days (standard deviation: 3.7)^13^, and the mean infectious period was assumed to be Erlang distributed with mean 2.9 days (2.1)^12^. A sensitivity analysis of these two parameters on the prediction performance of the SEIRD model was performed and can be found in the supplementary material S1.

For each country *k*, the daily transmission rate ($\beta _{t,k}$) and fatality rate ($\xi _{t,k}$) were modeled as a geometric random walk process, and sequential Monte Carlo simulation was used to infer the daily reproduction number, $R_{t,k}$ (defined as the transmission rate over the assumed incubation period $\beta _{t,k} /\gamma$), as well as the daily fatality rate. The remaining unknown parameters were estimated via grid search using the maximum likelihood method. In each country, we assumed the outbreak started from the first infectious case and that the entire population was initially susceptible. Further details on the model can be found in the supplementary material S1.

We summarized the patterns of estimated effective reproduction number using smoothed time series of $R_{t,k}$ generated by the Savitzky-Golay finite impulse response^14^ (FIR) filter. We compared hierarchical clustering from three algorithms: the WPGMA (Weighted Pair Group Method with Arithmetic Mean), the centroid linkage algorithm and the Ward variance minimization method.

Our study uses descriptive statistics to analyze the patterns of $R_{t,k}$ and early public health interventions before 15 May 2020. We characterized the time series of $R_{t,k}$ by the following metrics (we drop the country index *k* for simplicity):

- The days from the outbreak to the maximum of the estimated effective reproduction number curve (i.e. $R_{t}$ peak) before 15 May 2020;
- $R_{t}$ peak duration, defined as the time it takes $R_{t}$ to reduce to 50% of $R_{t}$ peak;

We related these metrics to three metrics captured over 8 social distancing policies:

- Policy timing (days from onset), defined as days from the outbreak onset to the intervention;
- Policy volume, which refers to the number of interventions applied; and
- Policy gap, defined as the average number of days between any two policies.

In addition to the static analysis of early control measures and $R_{t}$ patterns at the beginning of the pandemic, we conducted a time-lagged cross correlation (TLCC) analysis of the daily policy volume, daily effective reproduction number, and daily incidence counts from the first case detected in each country until the end of 2020. Please refer to the supplementary material S1 for implementation detail.

Lastly, we measured the prediction accuracy of our stochastic SEIRD compartment model up to 30 days from the time of prediction over 230 daily rolling windows ($j \in \{1,2,\ldots ,230\}$) from 15 May 2020 to 31 Dec 2020. An outbreak associated with prediction *j* in country *k* is defined according to:

$$\begin{aligned} Y_{k,j} = E_{k,j} / C_{k,j} > q, q \in [1,3], Y_{k,j} \in \{0,1\} \end{aligned}$$

where $E_{k,j}$ is the cumulative count of cases at the end of the *j*th window, $C_{k,j}$ is the cumulative count of cases at the beginning of the *j*th window, and *q* is the growth rate threshold. An outbreak is defined as $Y_{k,j}=1$.

To quantify the impact of the currency of transmission parameters on outbreak predictions, we estimated future incidence counts for each *j* and *k* in two ways: (1) with a constant $R_{t,k}$ defined as the average $R_{t,k}$ over the last five days at the beginning of the forecasting study (from the 11th to the 15th of May 2020), and (2) with a dynamic $R_{t,k}$ that is updated daily as the average $R_{t,k}$ over the last five-days before the time of prediction.

An Area under the Receiver Operating Characteristic Curve (AUROC) over the range of *q* was estimated using true positive and false positive rates^15^. The AUROC from the model with a dynamic effective reproduction number was compared against the AUROC from the model with a static effective reproduction number.

## Results

### Patterns of initial public health interventions and effective reproduction number

**Table 1:** Key descriptive statistics across clusters as at 15 May 2020.

	Metric	Cluster 1 (12)	Cluster 2 (20)	Cluster 3 (4)	Cluster 4 (51)	Cluster 5 (14)
$R_{t}$ Peak	Duration (days)	9.9 (2.14)	6.6 (1.38)	6 (1.52)	4.4 (0.56)	3.7 (1.12)
	Days from outbreak	14.7 (5.10)/45.7 (8.62)	21.8 (9.21)	30.3 (3.08)	25.5 (5.80)	18.1 (6.07)
$R_{t}$	Mean	2.3 (0.20)	2.7 (0.30)	2.9 (0.30)	2.6 (0.22)	4.8 (1.36)
	SD	1.4 (0.14)	2.5 (0.49)	2.8 (0.66)	3.1 (0.75)	12.7 (4.06)
Policy	Gap (days)	13.1 (3.97)	7.1 (2.04)	8.7 (5.75)	7.8 (1.93)	9.9 (2.59)
	Volume (number)	6.8 (0.50)	7.3 (0.62)	5.8 (2.63)	7.1 (0.34)	7.1 (0.45)
	Timing (days from onset)	41.8 (3.78)	12.9 (2.39)	34.5 (8.96)	11.2 (2.78)	7.9 (1.20)
Reported Statistics	Tests/’000	21.5 (8.95)	19.1 (10.93)	35.7 (13.1)	10.5 (6.5)	10.2 (7.21)
	Reported deaths/’000	0.13 (0.10)	0.08 (0.06)	0.56 (0.17)	0.01 (0.001)	0.13 (0.17)
	Reported infections/’000	1.5 (0.80)	1.7 (1.11)	4.1 (1.00)	0.9 (0.49)	2.1 (2.61)
	Death/Infection	0.06 (0.03)	0.04 (0.01)	0.14 (0.02)	0.03 (0.001)	0.05 (0.02)

This table displays selected descriptive statistics from 101 selected countries at the beginning of the pandemic up to 15 May 2020. The first section (as indicated by the solid horizontal lines) records the pattern of estimated daily reproduction number. Only cluster 1 is characterized by countries having two peaks of similar levels in their daily reproduction numbers. The second section summarizes the pattern of 8 social distancing policies. The last section records the average number of observed tests, reported deaths, reported infections per thousand population and reported deaths per infection. We averaged these values across countries in each cluster and recorded their sample standard error times the 95% confidence level value in brackets. For a detailed description of each examined policy the reader is referred to the supplementary materia S1l.

**Figure 2:**
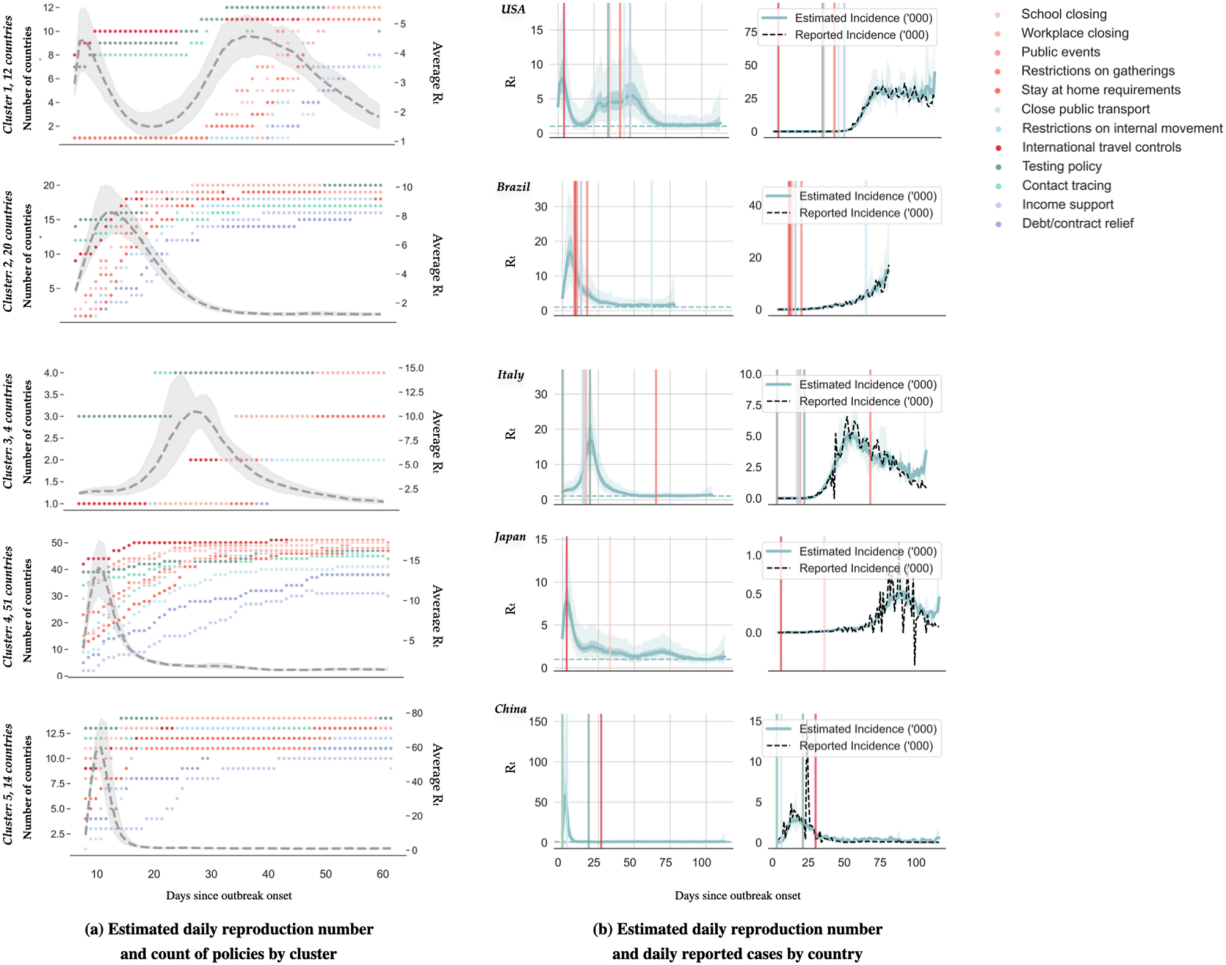
Patterns of early public health interventions and the evolution of effective reproduction number by cluster. (**a**) The left panel shows the estimated daily reproduction number for each cluster during the first 60 days of the epidemics. The dashed gray line represents the median and the light gray shading represents the 5th to 95th quantiles. The number of countries that adopted the corresponding selected public health intervention are displayed using colored dots. (**b**) The left side of the right panel shows the mean estimated daily reproduction number in selected countries until 15 May 2020. The right side of the right panel displays the reported (in dashed black line) and estimated average incidence counts (in solid blue line). In both cases, the light and dark blue shading represent 75% and 95% confidence intervals of the model estimate respectively, and the colored lines mark the initiation of the corresponding selected public health intervention.

Our three clustering algorithms identified the same 5 distinct patterns of $R_{t}$ at the second level of the hierarchy tree (results from WPGMA are provided in the supplementary material S1). Patterns of $R_{t}$ were summarized by their mean and standard deviation, peak duration, and days from outbreak to peak. Table 1 shows these values for each cluster, together with the timing, volume and gap of public health interventions, as well as the number of infections, deaths and tests. A visual illustration of the relationships among $R_{t}$, reported incidence and interventions can be found in Fig. 2.

Cluster 1 has 12 countries featuring the longest duration of $R_{t}$ peak. Interestingly, two $R_{t}$ peaks of similar levels were observed in these countries (please see Fig. 2), where the first one took place during the second week from the outbreak; and the second was after 45.7 (37.08, 54.25) days. This cluster includes countries such as Australia and US who started international travel control and contact tracing shortly after the initial outbreak, but delayed subsequent social distancing measures by a few weeks.

Likewise, cluster 2 had an average days from onset to $R_{t}$ peak of 21.8 (12.59, 31.01) days, which is about 7 days later than cluster 1. The average duration of $R_{t}$ peak in this cluster is also about 3 days shorter. The 20 countries in this cluster, such as Brazil and New Zealand, adopted most interventions (7.3 (6.68, 7.82)) with the shortest gap between any two interventions (7.1 (5.06, 9.05) days) compared to other clusters, and they have the second lowest mortality per infection (0.04 (0.033, 0.05) death/infection).

Cluster 3 contains 4 countries: Italy, Spain, Belgium and Sweden. The average level of $R_{t}$ and its duration are similar to those in cluster 2. However, their average mortality rate is more than 3 times higher than in the second cluster. This severe mortality burden has been featured in a recent study^16^. These 4 countries are also characterized by the highest infection rates (4.1 cases per thousand population), the highest testing rates (35.7 tests per thousand population), and an aging population (20.1% of the population of these four countries aged 65 or above^17^). These high infection rates are consistent with a low number of adopted interventions (5.8) and the time taken to apply the first social distancing policy (34.5 days).

Cluster 4 includes 51 countries that are similar in their transmission patterns, and cannot be distinguished under the second level of the hierarchy tree using the first 60 days of observations. The evolution of this cluster’s average $R_{t}$, including the timing and duration of its peak, is comparable to that of cluster 5 but with a much lower level and volatility. Meanwhile, this cluster’s policy adoption resembles that of cluster 2 but its $R_{t}$ peak duration is shorter.

Cluster 5 has 14 countries such as China, Iraq and Argentina. They had the highest average $R_{t}$ at 4.8 (3.44, 6.18), which was lifted by the $R_{t}$ peak during the initial outbreak (please see Fig. 2). However, their average peak duration is the shortest at 3.7 (2.58, 4.79) days. On average, these countries adopted their first social intervention 7.9 (6.7, 9.02) days after the outbreak, which is earlier than other clusters.

Overall, policy timing appeared to have a negative impact on the peak duration of $R_{t}$. For example, compare clusters 1 and 5, where the first has the longest transmission peak and the second has the shortest. This can be related to the timing of adopting social distancing measures, where cluster 5 only took one fifth of the time of cluster 1 to have around 7 policies in place. Across countries, the correlation between the number of adopted policies per unit time and the duration of the $R_{t}$ peak was − 0.26.

The gap between any two policies was also found to be correlated with the duration of the $R_{t}$ peak, with a correlation coefficient of 0.24. Clusters 1 and 3 adopted similar number of policies with similar delays. However, the average policy gap in cluster 1 was 5 days longer than in cluster 3.

There was not much difference in policy volume across clusters. By 15 May 2020, on average 7.0 (6.81, 7.29) social distancing measures had been adopted among all examined countries, and around 60% of them had both testing and contact tracing policies in place. Only 32% of these countries had income support and 51% had debt or contract relief policies. The pattern of adoption of each individual policy in these clusters is provided in the supplementary material S1.

The combined effect of less social distancing measures, and longer policy timing and gap can result in a prolonged period with a high level of $R_{t}$. By 15 May 2020, the average of median $R_{t}$ across examined countries had reduced from its peak of 20.5 (17.79, 23.20) to 1.3 (0.94, 1.74).

### Correlation among public health interventions, the effective reproduction number, and incidence counts

**Figure 3:**
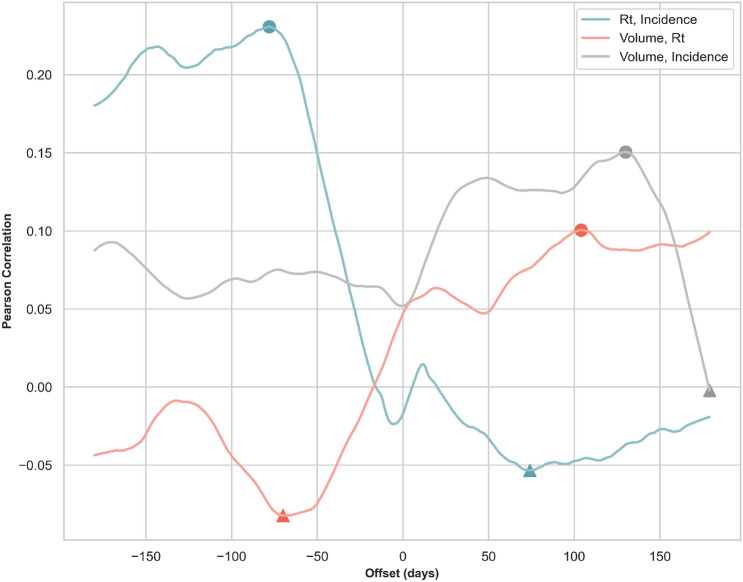
Time-lagged cross correlation (TLCC) among selected time series. This figure depicts TLCC among selected time series for an offset from − 180 to 180 days. The solid blue line shows the TLCC between the daily effective reproduction number and the daily reported incidence count. The solid red line shows the TLCC between the daily policy volume and the daily effective reproduction number. Finally, the solid gray line shows the TLCC between the daily policy volume and the daily incidence count. All calculations are averaged over the TLCC of each country from the first count until 31 Dec 2020. The filled circles and triangles indicate the peak correlations (the maximum positive correlation or the minimum negative correlation) between two series.

The time-lagged cross correlation (TLCC) among the daily effective reproduction number, daily incidence counts, and policy volume was estimated for each country using data from the first case detected in each country until 31 Dec 2020. Figure 3 shows the resulting correlation coefficients averaged over countries as a function of offset days.

As it can be seen in Fig. 3, the daily effective reproduction number leads the daily incidence counts by 78 days (that is when correlation is maximized). Meanwhile, the correlation between the policy volume and daily reproduction number is two-folded. On one hand, a higher policy volume leads to lower future $R_t$ (the negative correlation is minimized when $R_t$ lags the policy volume by 75 days). On the other hand, a lower $R_t$ is associated with lower future policy volume (the positive correlation is maximized when the $R_t$ leads by 102 days). Lastly, the negative association between the increased policy volume and declined incidence counts is small for lags up to 180 days. Instead, one can only observe that declined incidence counts are closely related to lower number of policies in the future (the positive correlation is maximized when the counts lead the volume by 135 days).

**Table 2:** Key descriptive statistics across clusters as at 15 May 2020.

	Cluster 1 (12)	Cluster 2 (20)	Cluster 3 (4)	Cluster 4 (51)	Cluster 5 (14)
$R_t$, Incidence	**0.34 (− 76)**	**0.4 (− 104)**	**0.24 (− 65)**	**0.21 (− 149)**	**0.39 (− 80)**
	− 0.15 (63)	− 0.09 (179)	− 0.19 (− 17)	− 0.04 (74)	− 0.04 (114)
Volume, $R_t$	0.13 (14)	0.21 (179)	0.17 (150)	**0.11 (103)**	**0.15 (160)**
	**− 0.16 (− 37)**	**− 0.23 (− 61)**	**− 0.29 (− 67)**	− 0.06 (− 90)	− 0.11 (− 78)
Volume, Incidence	**0.17 (− 105)**	**0.3 (102)**	0.19 (40)	**0.16 (132)**	**0.25 (168)**
	− 0.14 (− 2)	− 0.26 (− 129)	**− 0.21 (− 120)**	− 0.01 (− 179)	0.01 (− 90)

This table shows the maximum positive correlation and the minimum negative correlation among the daily policy volume, the daily effective reproduction number and the daily incidence count for each cluster identified in “Patterns of initial public health interventions and effective reproduction number”. We highlighted the correlation with the highest absolute value in each section and cluster. The values in the brackets record the offset days associated with the maximum or minimum correlation.

In Table 2, we present the TLCC peak correlations by cluster. The first section shows that the effective reproduction number is consistently leading the incidence counts by 65–149 days across clusters. The second section shows how policy volume leads the effective reproduction number in clusters 1–3 by 37–67 days, while in clusters 4 and 5, higher absolute correlations are observed when policy volume leads the effective reproduction number by 103 and 160 days respectively. The last section of the table records the relationship between policy volume and incidence counts. All clusters, except from cluster 3, show significant positive correlation between the two time series. In cluster 1, a greater absolute correlation is observed when policy volume lags incidence counts by 105 days, while in clusters 2, 4 and 5 policy volume leads incidence counts by 102–168 days respectively.

### Improved forecasting accuracy with a dynamic effective reproduction number

Forecasting of incident counts up to 30-days from the time of prediction was performed for each country. The model was able to identify 44 of the 53 countries which had a major outbreak (Defined by a growth rate threshold value $q > 1.5$, that is by the end of prediction window the cumulative counts would grow by $150\%$ compared to the counts at the start of the window.) 30-days from 15 May 2020 (true positive rate of $83.02\%$), as well as 38 out of 48 countries which did not experience an outbreak (true negative rate of $79.17\%$).

**Figure 4:**
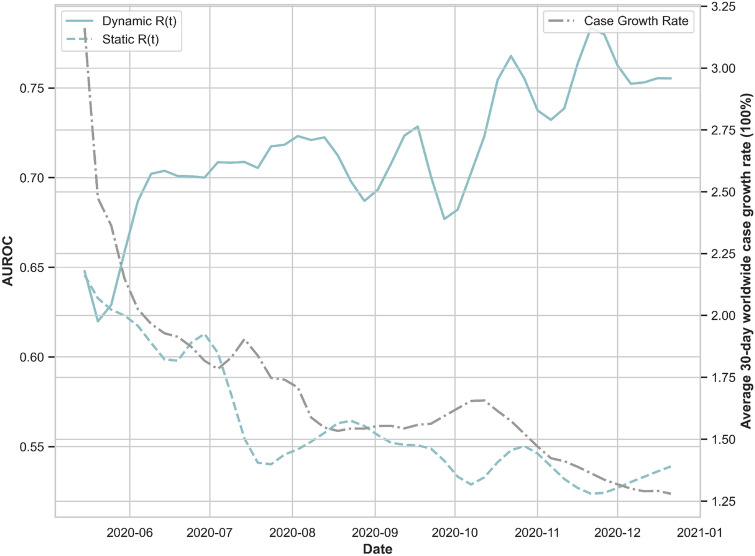
Outbreak prediction accuracy of the stochastic SEIRD model with dynamic and static effective reproduction numbers. This figure depicts the AUROC corresponding to 30-day outbreak predictions over 230 daily rolling windows from 15 May 2020 to 31 Dec 2020, and averaged over the selected 101 countries. The solid blue line corresponds to forecasts using the daily updated reproduction number averaged over the last five days previous to the time of prediction. The dashed blue line corresponds to forecasts in which the daily reproduction number remains static as the average over 11–15 May 2020.

Model predictions were reasonably stable to assumptions about the values of incubation period and the mean infectious period, although our sensitivity analysis (described in the supplementary material S1) observed an improvement in accuracy by assuming either a longer mean infectious period or a shorter incubation period.

Predictions were repeated over 230 daily rolling windows from 15 May 2020 until 31 Dec 2020. In the first experiment, $R_t$ is set constant as the estimated $R_t$ averaged over the previous 5 days. That is, when making a prediction in day 2, the model takes into account the updated reported counts, recoveries and deaths but $R_t$ remains the same as in day 1. In the second experiment, $R_t$ is updated dynamically every time we make a new prediction.

The AUROC averaged over all countries using these two methods is displayed in Fig. 4. As expected the dynamic update of $R_t$ produces more accurate results with $\text {AUROC} = 0.56\,\, (0.555, \,\, 0.568)$ for static $R_t$ vs. $\text {AUROC} = 0.72 \,\,(0.708,\,\, 0.723)$ for dynamic $R_t$.

## Discussion

To date, various algorithms have been applied to modeling the spatial and temporal dynamics of the transmission of SARS-CoV-2, including mathematical simulation-based models^2,4,5,7^ and statistical models^6^. For example, a Wuhan study^4^ used a deterministic, population-based compartment model. Baseline model parameters were fitted on observed local and exported cases. The impact of containment measures was explored by assuming various contact patterns given different physical distancing scenarios. The simulation suggested sustaining intense social distancing until the end of April 2020. Similarly, an Italian study^5^ used a more complex deterministic model to analyze the effect of social distancing. Initial parameters were inferred from fitting the model outputs to observed counts, deaths and recoveries while preserving a priori information on their relative magnitude. During the course of the simulation, the effect of social distancing was modeled by changing the value of key model parameters. They found predictions were extremely sensitive to key parameters related to disease transmission, which may be a problem in the presence of changing interventions. In this case, for instance, the basic reproduction number during 29 Mar to 6 Apr 2020 (when the national lock down was fully operational) was assumed to be 0.99, compared to our estimated effective reproduction number, which varied from $R_{t} =1.97\,\,(1.44,\,\, 2.50)$ to $1.07\,\, (0.76,\,\, 1.38)$ during that period. The difference between these numbers may be attributed to different model structures as well as to our consideration for the lag in reporting.

The assumption of population-wide homogeneous parameters limits the ability of these deterministic models to assess the spread of disease and its decline in relation to control measures^18,19^. During the current epidemic, a study in Singapore^7^ used agent-based influenza epidemic simulations to recreate a synthetic but realistic representation of the Singaporean population. The effect of distancing measures was then assessed by assuming different transmission rates under given social distancing policies. They recommended implementation of the quarantine of infected individuals, distancing in the workplace, and school closures immediately after having confirmed cases once international travel control has been imposed.

Further, Report 9 by Imperial College London^2^ assigned individuals to household, school, workplace and wider community and simulated scenarios by manipulating contact rates within and between groups. They found that a combination of case isolation, home quarantine and social distancing of aged population was the most effective scenario. This agrees with our findings that concentrated implementation of multiple interventions is most effective to contain transmission.

Rather than assessing the intervention impact via simulation, e statistical models aim to represent the empirical association between public health interventions and transmission rates and/or counts. For example, Report 13 by Imperial College London^6^ used a semi-mechanistic Bayesian hierarchical model to infer the impact of social distancing and travel lockdowns on the daily reproduction number in 11 European countries (11 countries in the original study are Austria, Belgium, Switzerland, Germany, Denmark, Spain, France, United Kingdom, Italy, Norway and Sweden.). The daily reproduction number was modeled as a function of the baseline reproduction number before any intervention together with multiplicative relative percent reductions in $R_{t}$ from interventions. Model parameters were fitted to observed deaths in these countries as a function of the number of infections. This model had heavy assumptions on prior distributions of model parameters and assumed consistent intervention impact across countries to leverage more data for fitting. By 28 Mar 2020, they found $R_{t}$ was reduced to around 1 across the 11 countries. Our study found similar reduction in $R_{t}$, which was sustained after one and a half months at 15 May 2020, when the average of the median of $R_{t}$ was 1.17 (0.91, 1.53) across these countries.

Our results highlight the importance of using real-time estimates of $R_{t}$ in these types of studies, since it is difficult to set epidemiological parameters under uncertain impact from public health interventions. This is particularly important given that prediction of future counts is very sensitive to simulation parameters. In this study, we demonstrated that updating the daily effective reproduction number facilitates more accurate simulations of future incidence counts as well as real-time monitoring of the effect of public health interventions.

We first conducted a hierarchical clustering analysis of $R_{t}$ from 29 Jan to 15 May 2020 in order to systematically characterize early public health interventions in relation to the patterns of disease transmission across countries. The analysis revealed significant heterogeneity in the effect of control measures on the daily effective reproduction number. For example, there were two transmission peaks in countries such as Australia and the US, which started international travel control shortly after the onset, but delayed subsequent social distancing measures by a few weeks. The clustering analysis also questioned the effect of testing and contact tracing in the presence of considerable community transmission and in the absence of social distancing. Italy, Spain, Belgium and Sweden were amongst the top 8 countries with the highest mortality burden^16^ and they were also amongst the top 10 countries carrying out most tests per population.

This heterogeneous effect makes it challenging to forecast future outbreaks by looking solely at the current interventions and disease counts. In fact, our time-lagged cross correlation analysis revealed that the negative correlation between the number of implemented polices and the level of virus transmission is reflected only in the updated daily effective reproduction number but not in the incidence counts in lags up to 180 days due to the substantial delay between the policy implementation and the reduction in incidence counts. Therefore, the calculated $R_{t}$ keeps a much closer track of the prevailing disease transmission trend than the reported incidence counts, and using updated $R_{t}$ in conventional SEIRD simulations can improve the predictions on future outbreaks.

Using the estimated daily effective reproduction number, we were able to forecast future outbreaks with reasonable accuracy from 15 May to the end of 2020. In particular, we predicted the worrisome increase in outbreak probability in countries such as Brazil in June 2020 and the possibility of second outbreaks in countries like USA, Italy and Japan in Nov 2020. We present the estimated $R_t$ and predicted incidence counts in these countries on a daily basis in the supplementary material S1 along with two most populous countries, China and India.

### Limitations

There are several limitations to our analysis. The estimated daily reproduction number is specific to our extended, population-level SEIRD model. This model does not represent the heterogeneity of transmission within a country, and therefore, may be less relevant for countries such as the US and China, which have varied adoption of interventions across regions. Researchers using other models need to estimate their own transmission parameters in a similar manner.

Our stochastic model does not fit well during the initial week of the epidemic outbreak, when only a small number of data points were available across countries. Over time, the availability and reliability of data improved, which was reflected in an increased model prediction accuracy, where the absolute bias of fitted daily incidents remains below 15% (please refer to the supplementary material S1 for more details). Nevertheless, we acknowledge that varied data quality across countries may influence the accuracy of our analyses. In particular, WHO’s daily Situation Reports shifted its reporting cutoff time on 18 Mar 2020, which compromised the comparability of its earlier figures. There was not much difference between the other two sources of disease counts^20^ except that Johns Hopkins also included estimates of presumptive positive cases that have been confirmed by state or local labs, but not by national labs. Two well-known problems of COVID-19 data are under-reporting of cases and delays in reporting. We attempted to account for both problems by introducing a parameter representing the proportion of actual cases detected via testing, as well as delays in reporting. We used plausible biological parameters based on current evidence, but these values might be refined as more clinical evidence becomes available. The standardized intervention measures from Oxford COVID-19 Government Response Tracker, may also suffer from inaccuracies. As the updating frequency of the tracker is performed once a week in most countries, we infer missing records of policies using the last recorded policy implementation.

## Conclusions

This study provides an estimate of the daily effective reproduction number of the SARS-CoV-2 transmission across countries and over time as various public health interventions were adopted. This allowed us to look at the evolution of disease transmission in the presence of containment measures. We confirmed the importance of using updated estimations of the reproduction number for the monitoring of future outbreaks.

## Supplementary Information

Supplementary Information
