# Supplementary material for: Evolution of disease transmission during the COVID-19 pandemic: patterns and determinants
Source: Sci Rep. 2021 May 26;11:11029. doi: 10.1038/s41598-021-90347-8 (PMC8155190; doi:10.1038/s41598-021-90347-8)
Supplement: Supplementary file 1 — Supplementary Information [file 41598_2021_90347_MOESM1_ESM.pdf]

# Supplementary Material: Evolution of disease transmission during the COVID-19 pandemic: patterns and determinants

Jie Zhu and Blanca Gallego

## SEIRD model calibration, fitting and prediction

Throughout the manuscript, we make use of publicly available data of newly confirmed daily cases, recoveries and deaths as reported by Johns Hopkins University (JHU) from 22 Jan until 31 Dec 2020. Our SEIRD model has been coded in Python 3.8 and a replicable example of the model can be found at github.com/EliotZhu/EpiModel.

We used an extension of the traditional SEIRD model that allows for variation in delays from onset to report and the detection rate of testing in each country. In this way, if $S_k, E_k, I_k, R_k$, and $D_k$ represent respectively the susceptible, exposed, infectious, recovered and dead for each country or region k, their change in time is modeled as:

$$dS_k(t) = -\frac{\beta_k(t)}{N_k(t)} S_k(t) I_k(t)\,dt \tag{1}$$

$$dE_k(t) = \frac{\beta_k(t)}{N_k(t)} S_k(t) I_k(t)\,dt - \sigma E_k(t)\,dt \tag{2}$$

$$dI_k(t) = \sigma E_k(t)\,dt - \gamma I_k(t)\,dt + \xi_k(t) I_k(t)\,dt \tag{3}$$

$$dR_k(t) = \gamma I_k(t)\,dt \tag{4}$$

$$dD_k(t) = \xi_k(t) I_k(t)\,dt \tag{5}$$

where $N_k(t) = S_k(t) + E_k(t) + I_k(t) + R_k(t) + D_k(t)$ is the total population after the travel ban if we ignore births and deaths unrelated to COVID-19.

The effective transmission rate, $\beta_k(t)$, *and* and the mean fatality rate, $\xi_k(t)$, for each country are assumed to follow geometric Brownian motions:

$$dlog\beta_k(t) = \beta_k(t)\,dB(t) \tag{6}$$

$$dlog\xi_k(t) = \xi_k(t)\,dB(t) \tag{7}$$

Meanwhile, the clinical parameters $\sigma$ - the mean rate of becoming symptomatic (that is 1/incubation period), and $\gamma$ - the mean rate of recovery (that is 1/infectious period) are considered constant and obtained from the literature with $\sigma = (5.2\ days)^{-1}$ and $\gamma = (2.9\ days)^{-1}$. We therefore implicitly assume individuals become infectious when they are symptomatic.

The unknown parameters of interest, $\beta_k(t)$ and $\xi_k(t)$, are estimated by jointly fitting the daily observed cumulative number of confirmed cases ($\widehat{C_k}$), confirmed recoveries ($\widehat{R_k}$) and deaths ($\widehat{D_k}$) using:

$$d\hat{C}(t) = \eta_k(t)\,\rho_k(\sigma E_k(t)\,dt - \gamma I_k(t)\,dt + \xi_k(t)\,I_k(t)\,dt), \tag{8}$$

$$d\hat{R}(t) = \eta_k(t)\rho_k\gamma I_k(t)\,dt, \text{and} \tag{9}$$

$$d\hat{D}(t) = \eta_k(t)\rho_k\xi_k(t)I_k(t)\,dt, \tag{10}$$

where $\rho_k$ represents a delay between the actual incidence, recovery or death date and their reported date, and $\eta_k(t)$, is the time-varying case detection rate.

The time-varying transmission rate, $\beta_k(t)$ and mortality rate, $\xi_k(t)$ were estimated using sequential Monte Carlo (SMC) by jointly fitting them to three data sets:

1. Daily number of new cases reported in each country by date from onset.

2. Daily number of recoveries reported in each country.

3. Daily number of deaths reported in each country.

The SMC process proposes trajectories of the unobserved state process according to the probabilistic rules specified in equations (1) to (10). For each country, we generated 300 trajectories $x_{n,j}$ with 5000 particles ($n = 1, 2, \ldots, 5000$, $j = 1, 2, \ldots, 300$) for $x = \beta_k(t)$ and $\xi_k(t)$ respectively. We first used the model outputs to calculate expected trajectories for each of the three datasets we are fitting to, then we used a negative binomial observation model to compute the likelihood of fitting to these datasets, using the expected values from model outputs. The time varying basic reproduction number can be calculated as $R_t = \beta(t)/\gamma$. The value of assumed delay from disease onset to reporting $\rho_k \in [0, 25]$, the detection rate at each time point $\eta_k(t) \in [0, 1]$, and the volatility of the random walks $\zeta_k \in [0, 1]$ were estimated using grid search via the maximum likelihood approach (see Figures 1 to 3). Initial expected values of $\beta_k(0)$ and $\xi_k(0)$ were set to zero.

Profile likelihoods (see Figure 4 ) for each parameter were constructed based on the joint likelihood distribution of fitted counts of incidence, recovery and death. We assumed that the outbreak started with one infectious individual in each country and that the country's population was initially fully susceptible. The accuracy of the fitting is presented in Figure 5.

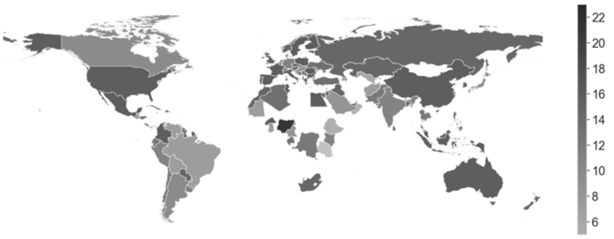


**Figure 1:** Assumed delay from disease onset to case reporting by country.
*Generated with GeoPandas 0.8.0 (geopandas.org)*

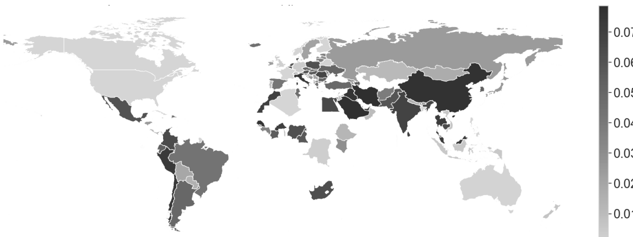


**Figure 2:** Assumed rate of undetected infection $(1 - \eta)$ by country.
*Generated with GeoPandas 0.8.0 (geopandas.org)*

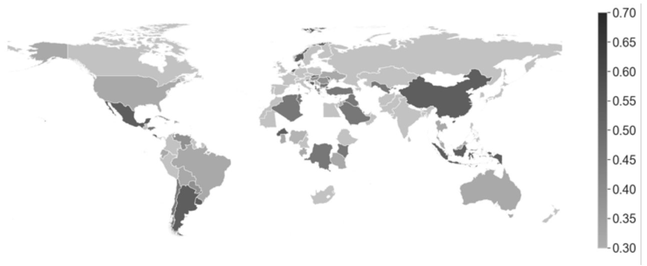


**Figure 3:** Assumed volatility of the transmission rate by country.
*Generated with GeoPandas 0.8.0 (geopandas.org)*

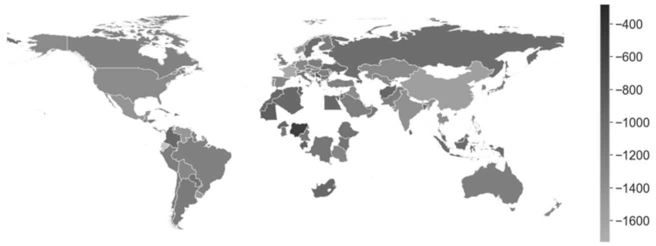


**Figure 4:** The joint likelihood profile of the fitted incidence counts, recoveries and deaths by country.
*Generated with GeoPandas 0.8.0 (geopandas.org)*

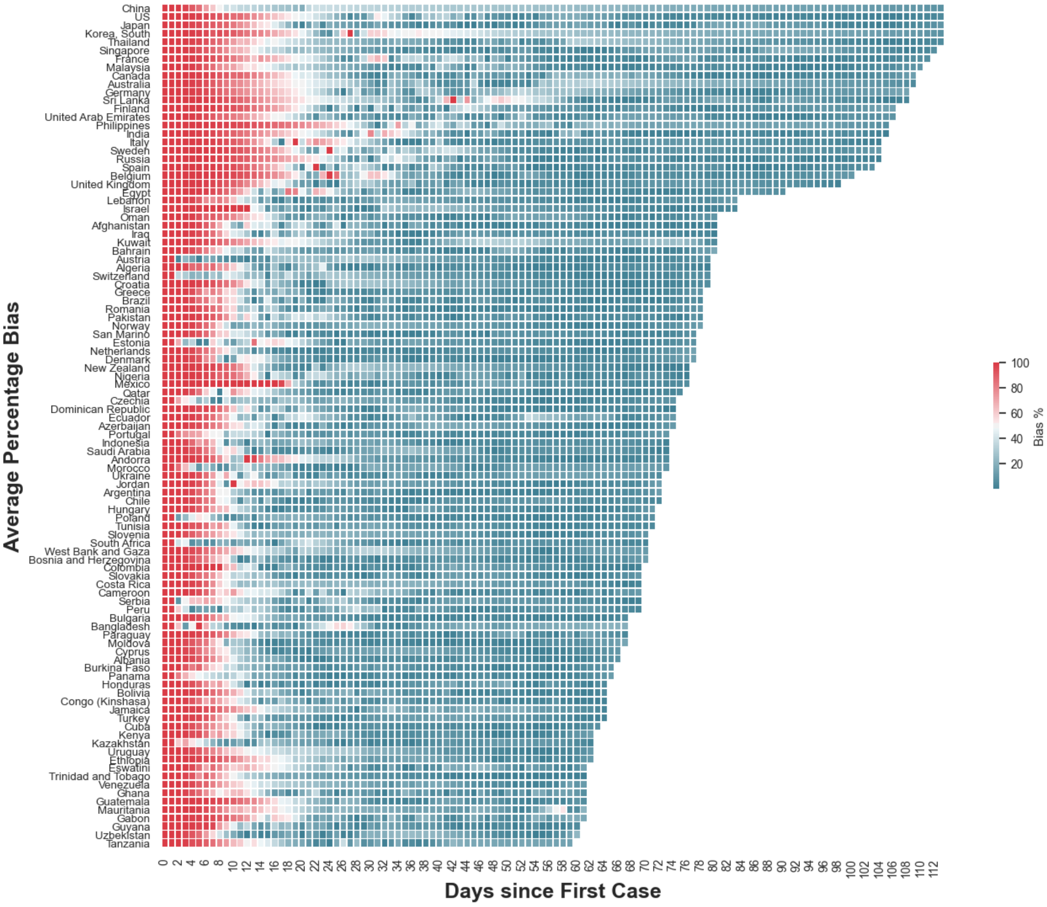


**Figure 5:** Percentage bias of fitted reported cases since outbreak onset in each country to 15 May 2020.

## Additional results for clustering analysis

We present the dendrogram from the WPGMA clustering algorithm in Figure 6. Clusters 1, 2, 3, 4, and 5 are colored as pale blue, light blue, blue, light gray and gray respectively. The effective reproduction number was fitted since the outbreak onset in each country for the first 60 days.

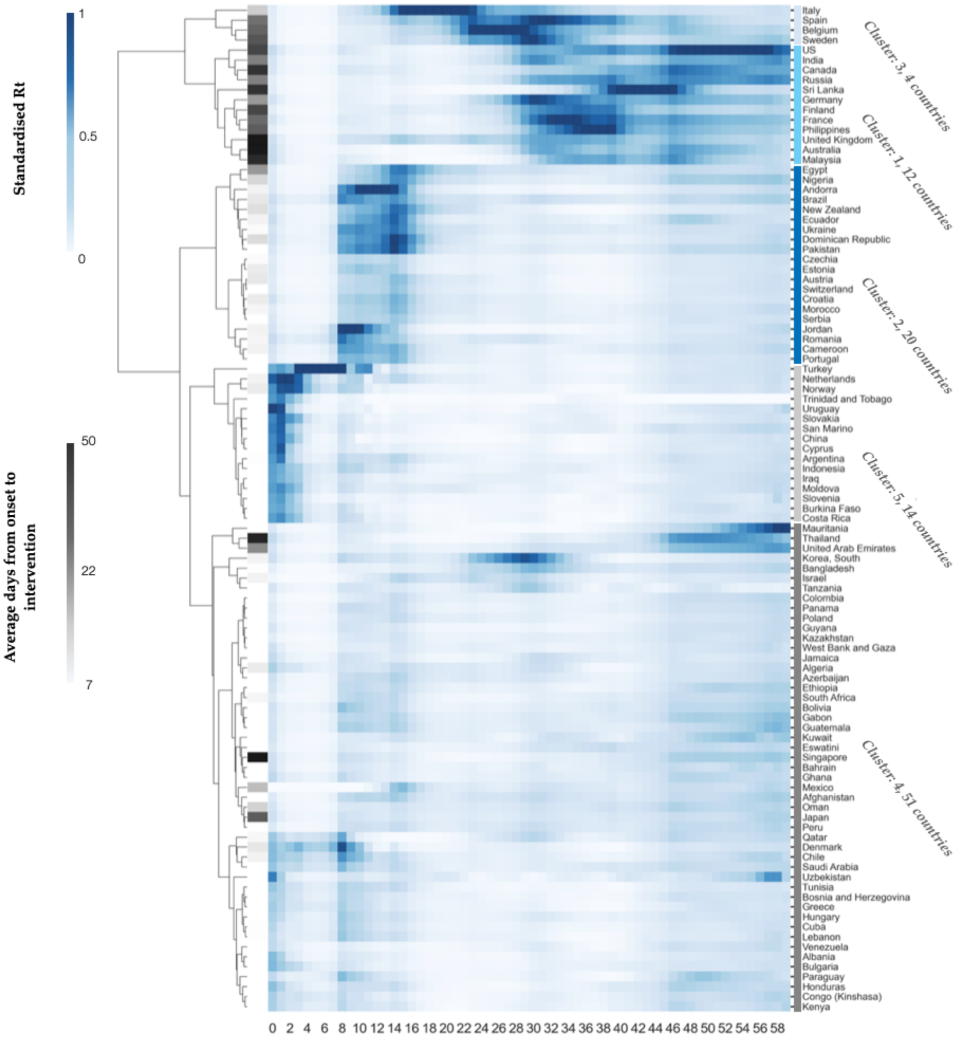


**Figure 6:** Dendrogram of the clustered time-series of estimated daily reproduction number by country and days since outbreak and countries.

Across the 101 selected countries, the estimated $R_t$ peaked on average 29·6 (25·36,33·81) days before the crest in infections. The average peak duration was 5·4 (4·84,6·06) days. The adoption of social distancing policies took place on average 15·6 (13,18·23) days from the first reported case and 5·3 (1·69, 8·84) days before the estimated $R_t$ peak. 76·9% of the examined interventions were applied before the end of peak duration. We report the descriptives in the Table 1.

**TABLE 1:** Supplementary descriptive statistics

| **Metric (% of policy adoption)** | **Overall (101)** | **Cluster 1 (12)** | **%** | **Cluster 2 (20)** | **%** | **Cluster 3 (4)** | **%** | **Cluster 4 (51)** | **%** | **Cluster 5 (14)** | **%** |
|---|---|---|---|---|---|---|---|---|---|---|---|
| Days from outbreak to $R_t$ peak | 26·3 (22·38,30·25) | 45·7 (37·08,54·25) | | 21·8 (12·59,31·01) | | 30·3 (27·22,33·28) | | 25·5 (19·7,31·24) | | 18·1 (12·03,24·25) | |
| Day from onset peak to $R_t$ peak | 29·6 (25·36,33·81) | 10·3 (1·75,18·92) | | 37·1 (31·02,43·08) | | 14·5 (4·3,24·7) | | 30·1 (23·72,36·55) | | 37·7 (26·66,48·77) | |
| $R_t$ peak duration (days) | 5·4 (4·84,6·06) | 9·9 (7·76,12·02) | | 6·6 (5·22,7·92) | | 6 (4·48,7·56) | | 4·4 (3·84,4·96) | | 3·7 (2·58,4·79) | |
| $R_t$ SD | 4·1 (3·15,5·08) | 1·4 (1·26,1·55) | | 2·5 (2·01,2·97) | | 2·8 (2·14,3·53) | | 3·1 (2·35,3·91) | | 12·7 (8·64,16·79) | |
| $R_t$ mean | 2·9 (2·63,3·17) | 2·3 (2·1,2·43) | | 2·7 (2·4,2·91) | | 2·9 (2·6,3·14) | | 2·6 (2·38,2·86) | | 4·8 (3·44,6·18) | |
| School closing (99%) | 17·1 (14·13,20·03) | 45·1 (40·38,49·79) | | 15·1 (12·53,17·67) | | 33 (23·13,42·87) | | 12·6 (9·08,16·22) | | 8·6 (6·79,10·5) | |
| | -9·2 (-12·63,-5·71) | -0·6 (-10·2,9·04) | 100% | -6·7 (-15·66,2·26) | 100% | 3·7 (-2·87,10·2) | 75% | -12·8 (-17·64,-8·01) | 100% | -9·5 (-16·06,-2·94) | 100% |
| Workplace closing (93%) | 23·7 (19·93,27·39) | 55·2 (46·54,63·79) | | 18·9 (16·06,21·64) | | 34·3 (22·13,46·54) | | 19·2 (14·75,23·59) | | 15·4 (6·84,23·93) | |
| | -1·9 (-6·01,2·13) | 9·5 (-3·7,22·7) | 100% | -3 (-11·44,5·54) | 100% | 5 (-4·26,14·26) | 75% | -5·2 (-10·88,0·45) | 90·2% | -0·9 (-11·94,10·1) | 92·86% |
| Cancel public events (96%) | 17·5 (14·63,20·4) | 46·8 (44·38,49·12) | | 14·8 (11·98,17·6) | | 33·3 (23·19,43·48) | | 13 (9·92,16·04) | | 8·6 (7·12,10·16) | |
| | -8·3 (-11·76,-4·75) | 1·1 (-7·78,9·95) | 100% | -7·3 (-16·65,2·13) | 95% | 4 (-2·88,10·88) | 75% | -11·3 (-16·29,-6·36) | 96·08% | -9·5 (-16·07,-2·93) | 100% |
| Restrictions on gatherings (93%) | 21·8 (18·47,25·19) | 48·9 (47·27,50·55) | | 16·5 (14,19) | | 41·3 (27·2,55·3) | | 17·4 (13·37,21·36) | | 16·8 (6·88,26·62) | |
| | -4·1 (-8·38,0·19) | 2·9 (-7·11,12·93) | 91·67% | -5·5 (-15·85,4·85) | 90% | 11 (-1·58,23·58) | 100% | -7·4 (-13·36,-1·41) | 96·08% | 0 (-13·03,13·03) | 85·71% |
| Close public transport (52%) | 23 (18·5,27·57) | 50·3 (44·1,56·57) | | 23·2 (18·74,27·72) | | 72 (0,0) | | 21 (14·28,27·79) | | 14·2 (11·96,16·48) | |
| | -3·3 (-9·5,2·97) | -5·7 (-32·21,20·88) | 25% | -2 (-16·13,12·13) | 65% | 46 (0,0) | 25% | -6·4 (-15·62,2·8) | 52·94% | -0·3 (-4·65,3·99) | 64·29% |
| Stay at home requirements (84%) | 25·8 (22·32,29·26) | 51·2 (45·57,56·88) | | 26·9 (20·36,33·52) | | 36 (23·25,48·75) | | 20·9 (16·57,25·29) | | 19·7 (13·63,25·82) | |
| | 0·3 (-4·24,4·88) | 5·3 (-7·89,18·55) | 75% | 4·4 (-8,16·89) | 90% | 6·7 (-2·82,16·16) | 75% | -2·8 (-8·95,3·36) | 86·27% | 0·2 (-10·24,10·6) | 78·57% |
| Restrictions on internal movement (88%) | 25·5 (22,29·08) | 51·1 (47·47,54·7) | | 21·8 (17·28,26·25) | | 30 (12·36,47·64) | | 21 (16·15,25·93) | | 21·8 (14·7,28·84) | |
| | -1 (-4·91,3) | 5·4 (-3·67,14·51) | 100% | -1·2 (-12·18,9·82) | 85% | 1 (-10·76,12·76) | 50% | -4 (-9·62,1·62) | 88·24% | 3·7 (-4·76,12·15) | 92·86% |
| International travel controls (99%) | 13·6 (11·2,15·9) | 20·5 (9·45,31·64) | | 14·1 (10·42,17·78) | | 34·5 (16·07,52·93) | | 10·8 (8·16,13·49) | | 11·2 (7·08,15·34) | |
| | -12·7 (-17·11,-8·37) | -26·6 (-40·12,-13·15) | 91·67% | -7·7 (-18·09,2·69) | 100% | 4·3 (-11·21,19·71) | 100% | -14·6 (-20·75,-8·54) | 100% | -6·9 (-15·33,1·47) | 100% |
| Testing policy (64%) | 33·8 (27·48,40·18) | 61·9 (44·41,79·39) | | 32·7 (23·24,42·13) | | 38·7 (13·84,63·49) | | 26·4 (16·83,35·88) | | 25·4 (13·79,36·96) | |
| | 6·8 (0·18,13·42) | 14·2 (-6·71,35·11) | 83·33% | 15·1 (4·15,25·97) | 80% | 9·3 (-15·08,33·74) | 75% | -0·6 (-11·32,10·04) | 54·9% | 6·1 (-8·67,20·92) | 57·14% |
| Contact tracing (60%) | 19·5 (14·02,25·03) | 26 (3·74,48·26) | | 15·9 (3·81,27·99) | | 10·8 (3·4,18·1) | | 20·6 (12·7,28·44) | | 17·9 (6·75,29·05) | |
| | -7·9 (-15·59,-0·15) | -18·1 (-43·75,7·53) | 75% | -9·2 (-31·36,12·96) | 50% | -19·5 (-26·31,-12·69) | 100% | -5·5 (-17·15,6·22) | 54·9% | 0·6 (-11·63,12·83) | 71·43% |
| Income support (32%) | 34·9 (29·53,40·29) | 51·8 (46·52,57·15) | | 23·7 (17·78,29·65) | | 39 (30·46,47·54) | | 36·5 (26·76,46·33) | | 24·2 (12·08,36·32) | |
| | 9·7 (4·14,15·3) | 9 (3·01,14·99) | 50% | 8·4 (0·58,16·28) | 35% | 7·3 (-2·29,16·96) | 75% | 14 (-0·6,28·6) | 21·57% | 4·4 (-4·99,13·79) | 35·71% |
| Debt/contract relief (51%) | 34·7 (28·75,40·71) | 58·8 (48·62,68·94) | | 33·7 (20·94,46·39) | | 45 (17·56,72·44) | | 31·6 (23·87,39·29) | | 10·5 (7·27,13·73) | |
| | 5·6 (-0·34,11·49) | 14·7 (7·61,21·72) | 75% | 14·4 (-3·16,32·05) | 45% | 14 (-15·4,43·4) | 50% | 3 (-5·26,11·18) | 50·98% | -12·8 (-27·6,1·93) | 42·86% |
| Policy gap (days) | 8·6 (7·34,9·84) | 13·1 (9·13,17·17) | | 7·1 (5·06,9·05) | | 8·7 (2·95,14·36) | | 7·8 (5·87,9·67) | | 9·9 (7·31,12·4) | |
| Policy volume (number) | 7 (6·81,7·29) | 6·8 (6·3,7·36) | | 7·3 (6·68,7·82) | | 5·8 (3·17,8·33) | | 7·1 (6·76,7·43) | | 7·1 (6·65,7·64) | |
| Policy timing (days) | 15·6 (13,18·23) | 41·8 (38·02,45·48) | | 12·9 (10·51,15·29) | | 34·5 (25·54,43·46) | | 11·2 (8·42,13·93) | | 7·9 (6·7,9·02) | |
| Tests/'000 | 14·4 (10·12,18·75) | 21·5 (12·55,30·37) | | 19·1 (8·17,30·02) | | 35·7 (22·6,48·89) | | 10·5 (4,16·9) | | 10·2 (2·99,17·36) | |
| Reported deaths/'000 | 0·08 (0·042,0·115) | 0·13 (0·027,0·228) | | 0·08 (0·018,0·145) | | 0·56 (0·388,0·736) | | 0·01 (0·008,0·019) | | 0·13 (-0·041,0·302) | |
| Reported infections/'000 | 1·4 (0·92,1·94) | 1·5 (0·7,2·39) | | 1·7 (0·59,2·71) | | 4·1 (3·1,5·03) | | 0·9 (0·41,1·41) | | 2·1 (-0·51,4·81) | |
| Death/Infection | 0·04 (0·037,0·051) | 0·06 (0·028,0·083) | | 0·04 (0·033,0·05) | | 0·14 (0·118,0·157) | | 0·03 (0·026,0·042) | | 0·05 (0·03,0·063) | |

This table records selected descriptive statistics on the early patterns of counts, recoveries, deaths, and public health interventions, as well as on the corresponding estimated effective reproduction number per cluster. All values were averaged over the countries in each cluster and include the 95% confidence interval in brackets. Clusters were estimated from patterns of the effective reproduction number in the selected 101 countries as at 15th of May 2020 as described in the main text of the manuscript. The first section of the table (as indicated by the solid horizontal lines) displays information on the estimated effective reproduction number. The second section of the table records the adoption of the 12 recorded social distancing and economic interventions. The % columns record the percentage of countries that implemented the corresponding intervention in each cluster. The last section displays the average number of observed tests, reported death, reported infections per thousand population and reported death per infection.

## The computation of time lagged cross correlation (TLCC)

To compute the time lagged cross correlation (TLCC) between two time series $A(t) = sin(t/100)$ and $B(t) = sin((t+1)/100)$, where $t \in \{0, 1, 2, \ldots, 1000\}$ (see Figure 7), the correlation between the two time series was repeatedly estimated after shifting the time series B by one time step at a time (blue line in Figure 7). The shifted time (namely the offset) at which the correlation is maximum was then calculated. For example, in the example represented in Figure 8, the correlation has a maximum when B is shifted by 100 time steps. Similarly, we can also shift B backwards in time and find that the correlation is minimum when B is shifted backwards by 100 time steps.

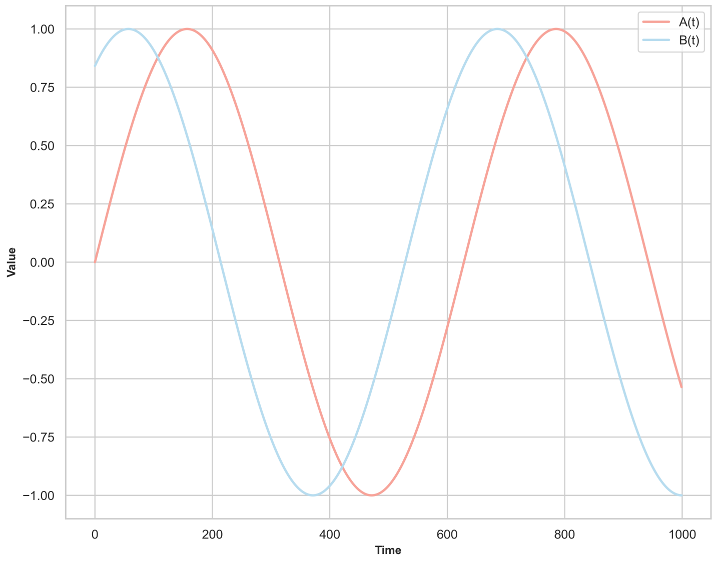


**Figure 7:** Example time-series

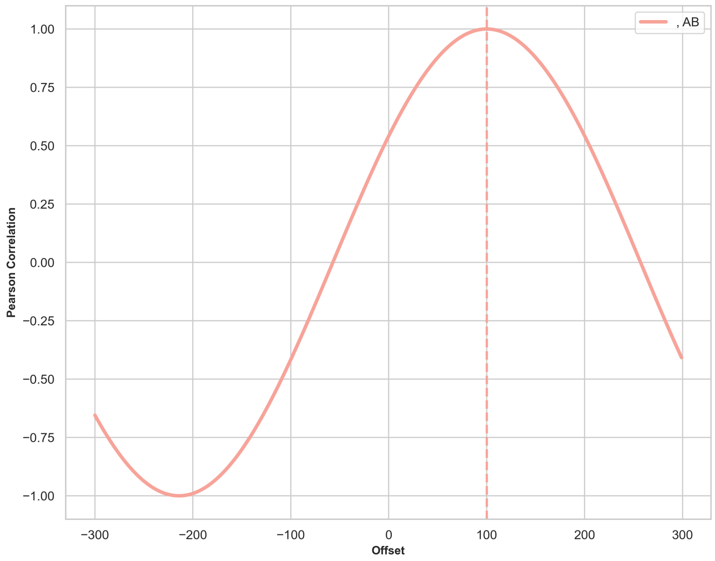


**Figure 8:** Example computation of time lagged cross correlation

## Prediction accuracy and sensitivity to key parameters

Prediction of future outbreaks (defined as a major growth in cumulative incidence count in a 30-day window with a growth rate threshold value $q \in [1, 3]$.) were conducted for each country and over 230 daily rolling windows from 15 May to 31 Dec 2020. The average AUROC by country (obtained by assuming different growth rate threshold) of this prediction can be found in Figure 9; its average by cluster is displayed in Table 2.

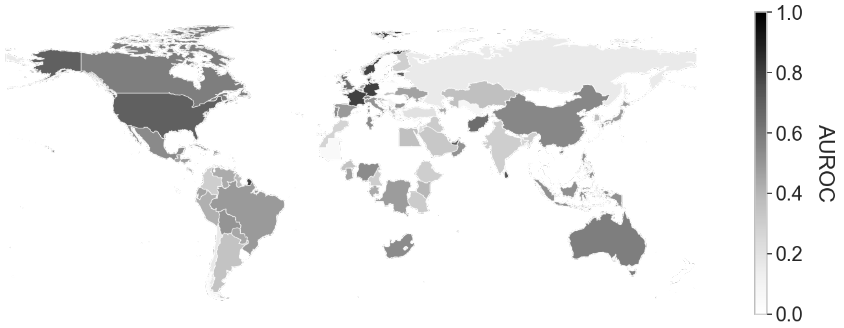


**Figure 9:** Outbreak prediction accuracy over 230 daily rolling windows of 30-days from 15 May 2020 by country. *Generated with GeoPandas 0.8.0 (geopandas.org)*

**Table 2:** Outbreak prediction accuracy by cluster

| | **Cluster 1 (12)** | **Cluster 2 (20)** | **Cluster 3 (4)** | **Cluster 4 (51)** | **Cluster 5 (14)** |
|---|---|---|---|---|---|
| AUROC (Dynamic $R_t$) | 0.69 (0.634, 0.741) | 0.63 (0.565, 0.687) | 0.55 (0.55, 0.55) | 0.61 (0.599, 0.625) | 0.70 (0.626, 0.775) |
| AUROC (Static $R_t$) | 0.57 (0.533,0.614) | 0.52 (0.473,0.564) | 0.55 (0.55, 0.55) | 0.51 (0.496,0.515) | 0.59 (0.529,0.641) |
| % of outbreaks | 33.7 (22.54, 44.88) | 36.6 (29.06, 44.26) | 30.6 (3.07, 58.26) | 30.7 (26.21, 35.28) | 37.1 (30.92, 43.35) |

This table records the outbreak prediction accuracy (AUROC) averaged per cluster. The values in the brackets report the 95% confidence interval of each estimations.

Across identified clusters in section 3.1 of the main manuscript, the prediction accuracy is best for clusters one and five (see Table 2).

To examine the sensitivity of the study findings to some model assumptions, we replicated the prediction study conducted in the main manuscript but assuming different levels of two key transmission parameters: the mean infectious period and the incubation period. We present the averaged AUROC over prediction windows and countries in Table 3. We observed an improvement in accuracy with longer mean infectious period or shorter incubation period.

**Table 3:** Prediction sensitivity

| **AUROC** | | $\sigma^{-1}$ | | | | |
|---|---|---|---|---|---|---|
| | | 3.8 | 4.5 | **5.2** | 5.9 | 6.6 |
| $\gamma^{-1}$ | 2.0 | 0.703 | 0.689 | 0.667 | 0.649 | 0.630 |
| | 2.5 | 0.733 | 0.709 | 0.708 | 0.676 | 0.652 |
| | **2.9** | 0.739 | 0.730 | 0.721 | 0.690 | 0.672 |
| | 3.2 | 0.756 | 0.737 | 0.727 | 0.712 | 0.687 |
| | 3.5 | 0.753 | 0.731 | 0.703 | 0.691 | 0.685 |

This table records the average outbreak prediction accuracy (AUROC) using dynamic $R_t$ for different values of $\gamma$: 1/mean infectious period, and $\sigma$: 1/mean incubation period.

In Figure 10, we present the estimated mean $R_t$ by six selected countries and the predicted new incidence counts since 15 May 2020 to 15 Jan 2021 using the proposed SERID model with dynamic $R_t$.

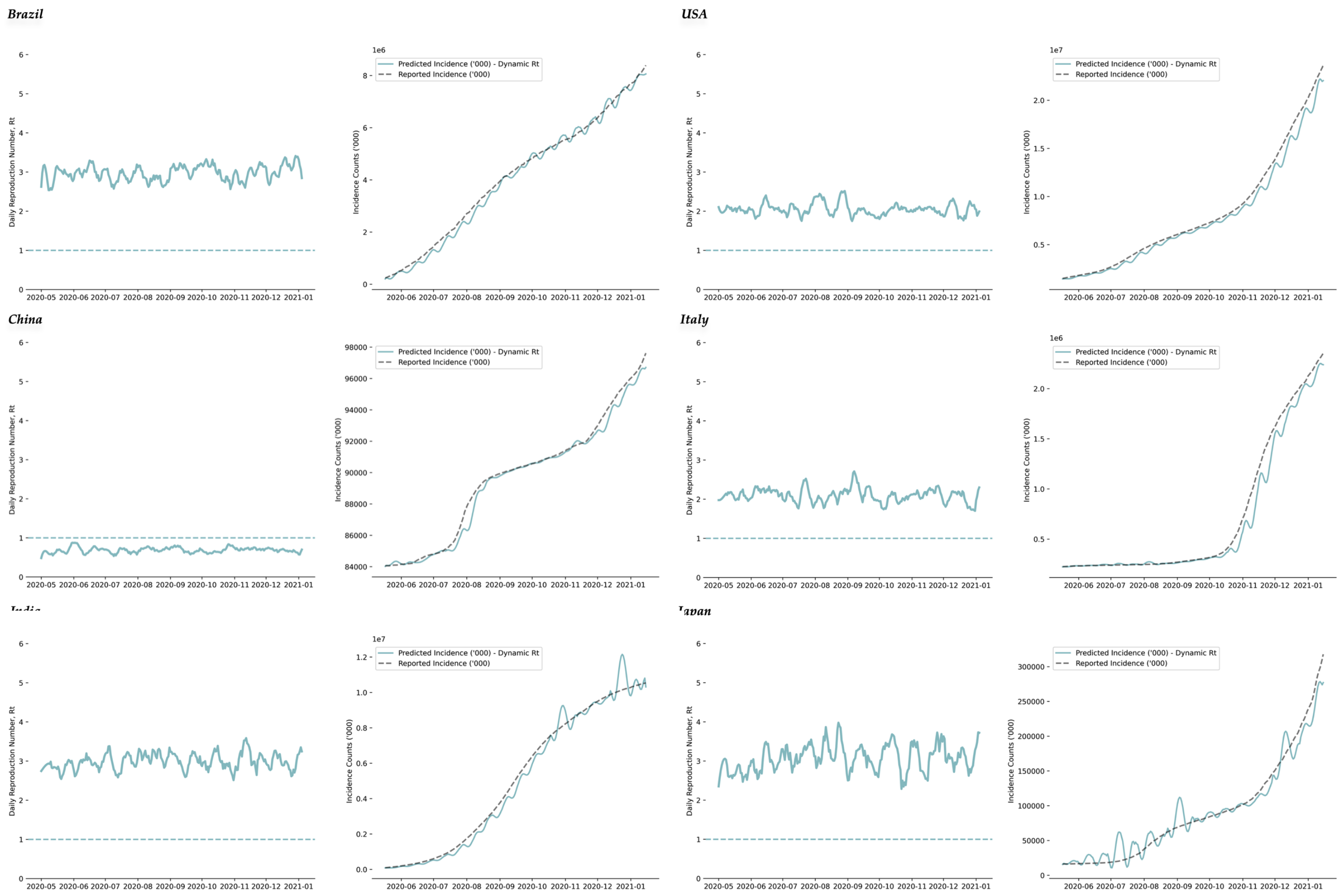


**Figure 10:** Estimated mean $R_t$ (left panel) and predicted incidence counts (right panel) by country since 15 May 2020 to 15 Jan 2021.
